# Acute Stroke Imaging: Recent Updates

**DOI:** 10.1155/2013/767212

**Published:** 2013-07-18

**Authors:** Prachi Dubey, Sachin Pandey, Gul Moonis

**Affiliations:** ^1^Department of Radiology, University of Massachusetts Medical School, Worcestor, MA 01655, USA; ^2^Department of Radiology, Beth Israel Deaconess Medical Center, Harvard Medical School, 330 Brookline Avenue, Boston, MA 02215, USA

## Abstract

Acute ischemic stroke imaging is one of the leading causes of death and disability worldwide. Neuroimaging plays a crucial role in early diagnosis and yields essential information regarding tissue integrity, a factor that remains a key therapeutic determinant. Given the widespread public health implications of stroke and central role of neuroimaging in overall management, acute stroke imaging remains a heavily debated, extensively researched, and rapidly evolving subject. There has been recent debate in the scientific community due to divided opinions on the use of CT perfusion and access-related limitations of MRI. In this paper we review and summarize recent updates relevant to acute stroke imaging and propose an imaging paradigm based on the recently available evidence.

## 1. Introduction

Acute ischemic stroke is one of the leading causes of mortality and morbidity worldwide. Statistics from the American Heart Association estimate an average of 1 stroke every 40 seconds in the United States amounting to approximately 795,000 people experiencing new or recurrent strokes, per year [1]. In view of the widespread public health impact of stroke and its profound impact on patients, stroke research has remained in the forefront. A recent systematic review article reported no significant difference between reperfusion strategies based on the current literature, emphasizing need for future randomized clinical trials to determine the efficacy of alternative reperfusion strategies [2]. As noted by Gonzales R in a recent commentary, the failure to recognize relative efficacy of treatment strategies can be partially attributed to lack of appropriate patient selection due to ineffective, inconsistent, and contradictory neuroimaging approach. This was one of the potential causes that lead to the halting of the Interventional Management of Stroke III Trial [3].

There is a critical need for reproducible and sensitive imaging biomarkers that allow accurate assessment of efficacy of rapidly evolving thrombolytic treatments. This underscores the primary need for standardization of imaging techniques across institutions so data from multicenter trials can be collectively analyzed. The glaring lack such consensus amongst imaging techniques was highlighted in a recent systematic review which found wide variability in the employed thresholds for CT and MR perfusion imaging and significant inconsistency in definitions of tissue states; factors which add to the widespread variability in perfusion-based assessment [4]. 

Despite the inherent challenges and past failures, stroke imaging is rapidly evolving with enormous ongoing research and global public health impact. In this paper we sought to review recent cumulative evidence including evolving expert opinions and recommendation to assess the adequacy of current state of clinical practices in acute stroke imaging. Based on our assessment we propose an optimal imaging paradigm for patients presenting with suspected acute ischemic stroke. 

## 2. Identification of Target Clinical Goals in the Acute Care Setting

The initial step in approaching the imaging paradigm is to summarize the targeted clinical goals for patients with suspected acute ischemic stroke in the acute care setting. The fundamental objective of treatment is to enable rapid reperfusion for maximal tissue salvation. There is substantial evidence to suggest efficacy of intravenous thrombolytic therapy in the first 4.5 hours from onset of symptoms as well as increased risk of hemorrhagic complications and lower efficacy outside the therapeutic window. The European Cooperative Acute Stroke Study (ECASS) investigators demonstrated the efficacy of treatment instituted within the first 4.5 hours [5]. This was confirmed on a systematic review with pooled data from 11 randomized controlled trials evaluating intravenous thrombolysis (IVT) and 3 randomized controlled trials evaluating intra-arterial thrombolysis (IAT). This review concluded efficacy of IVT within 4.5 hours of onset of symptoms, beyond which the risk of treatment outweighed the benefit. The clinical utility of expanding the treatment window to 6 hours with IAT treatment is currently investigational [6]. 

Based on the above considerations the following goals must be achieved to allow early initiation of treatment. The choice of imaging approach and interpretation protocol should be designed with the intent of addressing the primary clinical goals such as to allow safe and prompt initiation of thrombolytic strategies. 

### 2.1. Goal 1

*Exclusion of Primary Intracranial Hemorrhage, Assessing for Alternate Etiologies for Symptoms and Treatment Contraindications.* Once the patient presents to the ER with neurological symptoms possibly corresponding to a suspected acute stroke, the initial goal is a “rule out” approach. This is particularly critical in those patients with early presentation, as they are most likely to benefit from rapid institution of reperfusion therapy.

Initial evaluation focuses on exclusion of primary intracerebral hemorrhage (PICH), intracranial metastasis, tumor with herniation, or other alternate etiologies explaining the clinical picture. 

### 2.2. Goal 2

*Infarct Characterization: Identifying of Core, Quantification of Core Volume, Imaging of Penumbra and Pial Collateral Vessels*. This has been thoroughly reviewed and concisely presented as the “core, clot, collateral” approach and is currently the mainstay of acute stroke neuroimaging [7]. 

### 2.3. Nonenhanced Head CT

Theoretically Goals 1 and 2 can be assessed on NECT/CTA combination, which is the most ideal single-step imaging solution. Guidelines published by American Heart Association and American Stroke Association Stroke Council in 2007 mandate universal and immediate availability of nonenhanced head CT (NECT) within 30 minutes of initial presentation to the ER [8]. 

There is no controversy regarding the utility of NECT with regards to accomplishing Goal 1. In particular it is widely accepted that NECT can reliably exclude intracranial hemorrhage, which is critical for therapeutic decision making. 

**Figure 1:**
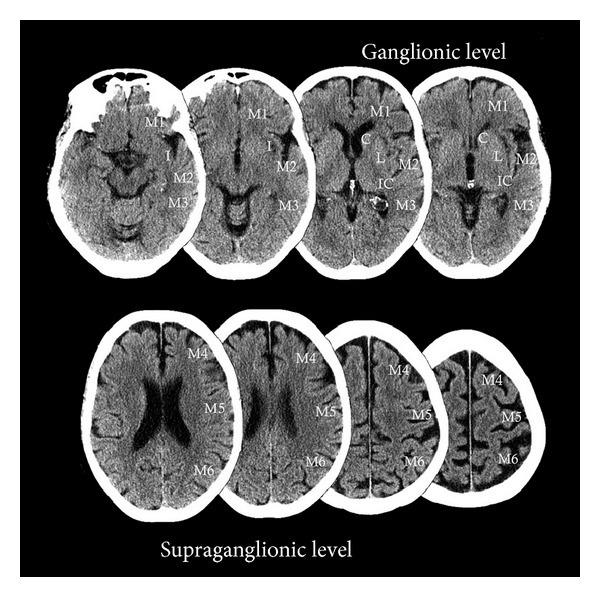
Obtained from http://www.aspectsinstroke.com/, demonstrating the ASPECTS scoring methodology, axial NCCT images showing the MCA territory regions as defined by ASPECTS. C, caudate, I, insularribbon, IC, internal Capsule, L, lentiform nucleus, M1, anterior MCAcortex, M2, MCA cortex lateral to the insular ribbon, M3, posteriorMCA cortex, M4, M5, M6 are the anterior, lateral, and posterior MCAterritories immediately superior to M1, M2, and M3, rostral to basal ganglia. Subcortical structures are allotted 3 points (C, L, and IC). MCA cortex is allotted 7 points (insular cortex, M1, M2, M3, M4, M5, and M6). (Reprint permission obtained from Dr. Mayank Goyal, professor of radiology and clinical neurosciences, * *Foothills Medical Centre, * *University of Calgary).

In terms of infarct characterization to address Goal 2, NECT-based scoring system designed by the Alberta Stroke Program, commonly referred to as the ASPECTS scoring system, (Alberta Stroke Program Early CT Score) provides an effective tool for quantifying early ischemic changes in the MCA territory (Figure 1). This methodology has provided useful prognostic information with regards to response to reperfusion. Patients with high ASPECTS scores (8–10) corresponding to low infarct volume on initial imaging demonstrated the best clinical outcomes [9]. However, as noted by Demchuck et al. the greatest limitation of ASPECTS application is the inability to accurately visualize the early ischemic changes on NECT in the real world setting, [7]. This is more problematic in the setting of preexisting white matter changes. An interobserver reliability study demonstrated a 77% concordance for total ASPECTS score with lower agreement for scores based on a cut-off (>7 and ≤7) and also lower agreement for cortical and internal capsule regions [10]. Overall, there is substantial cumulative evidence indicating ASPECTS NECT scoring as a very objective, semi-quantitative, prognostic tool and we recommend utilization of online resources to aid ASPECTS utilization in early acute stroke imaging; see http://www.aspectsinstroke.com/ [11].

### 2.4. MRI with Diffusion-Weighted Imaging

There is conclusive evidence regarding the sensitivity of MRI with diffusion-weighted sequences for the detection of infarct core including those cases in which the infarct core remains occult on standard T2-weighted imaging [12]. Evidence-based guidelines proposed by the Therapeutics and Technology Assessment subcommittee of the American Academy of Neurology, endorses the role of DWI in accurate diagnosis of acute ischemic stroke particularly in the first 12 hours as being superior to NECT and also suggest, that baseline DWI infarct volume has predictive ability towards final infarct volume and overall clinical outcomes [13]. 

Recent considerations for the same were reviewed by R. Gonzalez indicating a significant role of MRI with diffusion-weighted sequences in identifying infarct core and allowing assessment of core volume, which is a useful predictor of treatment efficacy [14]. In the past, pretreatment infarct core volume has been demonstrated to be a highly specific predictor for malignant middle cerebral artery infarction at a threshold core volume of greater than 82 mL. [15]. The MR Stroke study group investigators found a 5.8 fold increased risk of symptomatic intracerebral hemorrhage in patients with large-volume infarct cores (>100 mL) compared to small (<10 mL) and moderate (10–100 mL) ones [16]. A core volume threshold of approximately 70 mL has been suggested as having dichotomous prognostic implications with patients with higher core volumes having unfavorable outcomes regardless of treatment [17, 18].

A recent study demonstrated that posttreatment final infarct volume (FIV) also has significant influence on clinical outcome in patients undergoing IAT [19]. This study showed high specificity for poor outcome with FIV of >90 cm^3^. Therefore with regards to established management goals, MR with diffusion-weighted imaging provides the most accurate information related to Goal 2 principles of core identification and core volume quantification. However, diffusion-weighted imaging alone has limited utility for detection of penumbra for which MR perfusion estimates are more reliable and it provides little information about vascular substrates, including assessment of occluded vessel and degree of pial collateralization.

Additionally, there are accessibility issues due to individual contraindications to MR and limited availability of MRI leading to underutilization of MRI in emergent setting. A recent study evaluated the adherence to AAN guidelines of preferring MR over CT in the initial 12 hours of presentation and revealed that the target was met in less than 1/3 of patients in their study [20]. 

### 2.5. Mismatch Imaging: CT Perfusion and MR Perfusion

 Perfusion imaging either CT or MR is most relevant in terms of ability to delineate the ischemic penumbra. The clinical utility of penumbra imaging has long remained an issue of debate. The hypothesis of penumbra identification is that identification of “at-risk” tissue may allow widening of the treatment window beyond 4.5 hours and allow detection of patients who will either benefit from treatment or those in whom treatment is not likely to cause improved outcome. 

It is notable that the recent study by the MR Rescue investigators found no role of penumbra imaging in selecting patients likely to benefit from endovascular therapy within 8 hours from onset of symptoms. There was evidence of good functional outcome in patients with favorable penumbral pattern in the late time window regardless of recanalization. Interestingly, this study raises the possibility that patients who have a favorable penumbral pattern may be inherently more resilient to the effects of vascular occlusion and therefore harbor a favorable outcome regardless of treatment, thus explaining lack of differential effect of therapy when stratified on the basis of penumbral pattern [21]. 

Nevertheless there remains an interest in imaging penumbra due to its potential role as a prognostic biomarker. Previously, CT perfusion performed soon after the initial NECT has been supported as being a safe and efficacious strategy for imaging tissue at risk [22]. On the other hand a recent study demonstrated low sensitivity of MTT maps in predicting acute infarct detectable on DWI sequence. CBV also did not correlate with the DWI abnormalities. This study advocates against utilization of CTP in acute NVS [23].

Reliance on postprocessing, restricted brain coverage, and vendor related-differences in processing algorithm are primary limitations which have not yet been completely addressed and remain as mitigating factors in enabling wider utility of CTP. Additionally the inherent low contrast to noise ratio increases susceptibility to artifacts and lowers overall sensitivity. These considerations were reviewed by R. Gonzales and for the same reasons the clinical utility of CTP was felt to be doubtful in the current state of practice [3]. 

 On a contrary note, a recent expert commentary by M. Lev acknowledges the aforementioned CTP limitations but continues to endorse this method due to its relative cost efficacy, rapid availability, and potential for quantitative assessment relative to MRI [24]. A recent study demonstrated that CTP-based penumbra volume was an independent predictor of clinical outcome in 90 days along with recanalization status. This study also demonstrated that the CT perfusion based penumbra volume could not be accurately assessed by clinical parameters, NECT or CTA [25]. CTP rCBF maps with appropriate threshold levels are felt to represent an accurate estimate of the infarct core [26, 27]. 

MR perfusion parameters are equally sensitive in depicting tissue at risk although expense, lack of universal applicability and access issues in the ER setting remain limiting factors. Recently the applicability of MR Perfusion was reviewed by M. Fisher, highlighting its utility in delineating tissue at risk of infarction and thus holding promise in expansion of the therapeutic window. A key aspect underscored in this review relates to the identification of “benign oligemia,” which refers to hypo perfused tissue which will not proceed to infarction regardless of treatment. The MR perfusion parameter *T*_max⁡_ with *T*_max⁡_ delay of >5 to 6 seconds compared to normally perfused tissue was considered to be a useful indicator of impending infarction in the absence of reperfusion [28]. Using pooled data from the DEFUSE (diffusion and perfusion imaging evaluation for understanding stroke evolution) and EPITHET (echoplanar imaging thrombolytic evaluation trial) studies, Mlynash et al. found that based on *T*_max⁡_ and diffusion-determined mismatch, patients with a mismatch who have large core volumes (size of DWI lesion) or large perfusion defect (large-volume, severe *T*_max⁡_ delay) had unfavorable outcomes despite reperfusion. This study suggested a *T*_max⁡_ > 8 secs with a volume of approximately 100 mL as an adequate threshold for identification of patients with malignant profile of infarction who would be poor candidates for reperfusion therapy [29].

### 2.6. CT Angiography

**Figure 2:**
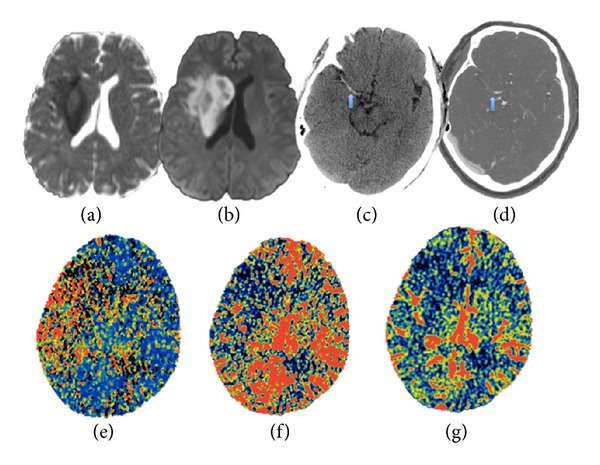
(a) and (b) ADC map and DWI map with restricted diffusion in the setting of cytotoxic edema from acute ischemic infarct in right MCA territory. (c) NECT showing hyperdense right MCA compatible with acute thrombosis. (d) CTA image with thrombosis in the corresponding segment of right MCA. (e), (f), and (g) CTP with elevated MTT, reduced cerebral blood flow, and blood volume in the right MCA territory.

CT angiography continues to be the superior method for characterization of vascular anatomy.  Figure 2 in addition to allowing for assessment of the site of vascular occlusion, it also allows assessment of the presence of calcifications and atherosclerotic disease, which can influence recanalization techniques. It is also the most effective non-invasive means of assessment of leptomeningeal collaterals, which not only determines rate of core expansion but also influences possibility of hemorrhagic transformation [30]. A study by Bang et al. demonstrated higher risk of hemorrhagic transformation in patients with poor collateral status [31]. Another study demonstrated discriminatory ability of a poor collateral score in detecting a malignant profile (larger DWI lesion volume at baseline, higher median NIHSS and functional dependency at 3 months after stroke) [32].

A recent study showed that CTA evidence of occlusion of distal internal carotid, proximal middle cerebral, or basilar arteries as a predictor of poor outcome and added incremental predictive value to NIHSS. This suggests the utility of CTA in early phase treatment decision making [33].

A potential confounding factor is acquisition protocol-dependent overestimation of infarct core volume using CTA source images for detecting of early ischemic changes [34]. The source images acquired by slow CT acquisition demonstrated greater infarct volume correlation with MR diffusion core estimates compared to the multislice CT scanner. This overestimation has critical clinical implications given it may prevent institution of reperfusion treatments in patients who could have potentially benefited from reperfusion therapy [34].

Overall, CTA has a definite prognostic role in acute phase of stroke imaging. In particular, with relevance to our defined Goal 2, acute-phase CTA can enable assessment of site of occlusion, integrity of vessels in terms of atherosclerotic disease, and degree of collateral flow, all of which influence management decision making (Figure 2). It must, however, be kept in mind that CTA entails exposure to radiation and iodinated contrast, which are potential pitfalls of utilization.

### 2.7. Proposed Imaging Paradigm

Despite significant advances in our understanding of physiologic surrogates of imaging observations and the respective technical confounds, there are still considerable debate and lack of consensus particularly with relevance to penumbra imaging and role of perfusion imaging as it relates to core characterization and penumbra estimation. 

If we take a minimalist approach, the expert opinion seems to converge most definitively on two standard queries prior to therapeutic decision making (1) is there primary intracranial hemorrhage? and (2) what is the volume of the infarct core? These two components combined with clinical neurological assessment seem to be most directly related to clinical outcome in postperfusion recovery phase and will help stratify patients appropriately for treatment decision-making. 

The limitation of the minimalistic approach is that although it raises specificity, by helping us identify those that will have a favorable outcome after-reperfusion, it also at the same time lowers sensitivity, thereby potentially excluding patients who may have benefitted from more aggressive reperfusion therapy.

One of the ways to achieve efficient imaging selection for treatment triage is development of a unimodal imaging protocol. Simplistically stating, a one-stop, all-inclusive imaging protocol can accurately and reproducibly classify patients who will either (a) benefit from treatment or (b) have no impact or negative impact of treatment. NECT is the most obvious choice for Goal 1-related aspects of management. However, beyond that it would be ideal if the CT imaging and interpretation protocols can be optimized in such a way that Goal 2 can be consistently and reliably achieved in acute-phase urgent care setting on the same CT scanner without having to transfer the patient. We believe that in many cases this is feasible, particularly when the ASPECTS score is carefully interpreted, providing infarct core volume information. To assist in the latter, we encourage the use of online resource described by Modi et al. [11] (http://www.aspectsinstroke.com/) to optimize utilization of NECT. This can be followed by CTA/CTP to assess the remaining aspects of Goal 2 with relevance to site of occlusion, collateral flow assessment, and penumbra imaging, all of which have been established as either predictors of treatment efficacy or overall clinical prognosis in poststroke recovery phase. This approach minimizes scanner time and utilizes the most widely available technologies only. Based on the considerations presented in the Gonzalez and M. Lev commentaries, we agree that NECT with ASPECTS assessment along with CTA can provide sufficient information for adequate triage of patients who will benefit from treatment. The remainder of the imaging including penumbra imaging with CTP can be performed while the treatment implementation has begun minimizing the “door-to-needle” time [3, 24]. 

However, we acknowledge coexistent evidence that indicates limitation of NECT in core characterization due to multitude of factors including inherently low sensitivity to early ischemic changes. It is agreed upon that core volume is a key determinant of treatment efficacy, for which MR with DWI is the imaging gold standard. In an ideal world with no cost, availability or individual applicability issues, an all inclusive MR protocol for acute stroke imaging would be more preferred from the viewpoint of obtaining accurate and consistent tissue specific information with minimum susceptibility to postprocessing variability. 

As stated above we emphasize that if MRI is not available and decision for endovascular therapy has to been taken based on the initial NECT, then immediate CTA (to assess vessel occlusion and collateral status) and CTP (infarct core assessment on rCBF map) should be considered (Figure 2). This approach would require less than 10 minutes and can be performed while the intravenous thrombolytic agent is being initiated [3, 24]. 

The utility of mismatch imaging is undoubtedly promising; however, the recent body of evidence does not provide compelling arguments to necessitate a paradigm shift particularly in routine clinical settings outside of major academic institutions. This is especially true in light of the results from the recent penumbra-based trial of imaging selection by the MR Rescue investigators demonstrating no utility of penumbra imaging in detecting patients who would benefit from endovascular therapy of acute ischemic stroke [21]. 

At this time with proven efficacy of IVT within the first 4.5 hours, it is notable that the frequency of thrombolytic therapy in patients with acute ischemic stroke remains remarkably low. A large multicenter study found only 3% utilization of thrombolysis for all acute ischemic stroke patients and only 10% for those who presented within the first 3 hours [35]. This is largely related to complexity of imaging protocols and a general lack of consensus amongst the experts regarding imaging appropriateness leading to inability to select the appropriate patients who would benefit from treatment in a timely fashion. In light of these compelling statistics, we strongly encourage a minimalist imaging approach, which can be reliably and consistently reproduced regardless of variability in institutional capabilities. NECT with ASPECT emphasis with simultaneous CTA/CTP and prompt MR with diffusion remain the most rigorously optimized imaging tools.
